# Frequency of infections in 948 MPN patients: a prospective multicenter patient-reported pilot study

**DOI:** 10.1038/s41375-020-0890-1

**Published:** 2020-05-30

**Authors:** Carl C. Crodel, Kathleen Jentsch-Ullrich, Steffen Koschmieder, Dietrich Kämpfe, Martin Griesshammer, Konstanze Döhner, Philipp J. Jost, Denise Wolleschak, Susanne Isfort, Frank Stegelmann, Stefanie Jilg, Verena Hofmann, Guiseppe Auteri, Tobias Rachow, Philipp Ernst, Annamaria Brioli, Marie von Lilienfeld-Toal, Andreas Hochhaus, Francesca Palandri, Florian H. Heidel

**Affiliations:** 1 Innere Medizin 2, Hämatologie und Onkologie, Universitätsklinikum Jena, Jena, Germany; 2 Gemeinschaftspraxis Hämatologie und Onkologie, Magdeburg, Germany; 3 Department of Hematology, Oncology, Hemostaseology, and Stem Cell Transplantation, Faculty of Medicine, RWTH Aachen University, Aachen, Germany; 4 Germany Study Group MPN, GSG-MPN, Germany; 5 Praxis Hämatologie und Onkologie, Lüneburg, Germany; 6 OSHO Study Group—Working Party MPN, WP-CMPN, Germany; 7 Johannes Wesling Klinikum, Universitätsklinikum für Onkologie, Hämostaseologie und Palliativmedizin, UKRUB, Universität Bochum, Minden, Germany; 8 Innere Medizin III, Universitätsklinikum Ulm, Ulm, Germany; 9 Medizinische Klinik 3, Hämatologie, Onkologie, Klinikum rechts der Isar, Technische Universität München, München, Germany; 10 Klinik für Hämatologie und Onkologie, Universitätsklinikum Magdeburg, Magdeburg, Germany; 11 Praxis für Hämatologie und Onkologie, Erding, Germany; 12 Institute of Hematology and Clinical Oncology “L. and A. Seràgnoli” Sant’Orsola-Malpighi University Hospital, Bologna, Italy

**Keywords:** Myeloproliferative disease, Risk factors

## To the Editor:

Recently, several studies have reported on immunosuppression and infectious complications in patients with myeloproliferative neoplasms (MPN) receiving Janus kinase (JAK) inhibitor therapy (reviewed in [1]). These datasets included analyses of large multicenter trials comparing JAK inhibitor therapy with best available therapy or placebo control. Specific infections such as herpes virus reactivation appear to be more prevalent in patients treated with the JAK inhibitor ruxolitinib (RUX) and opportunistic infections have been described also outside of clinical trials [1]. However, it is not known to which extent the underlying malignancy (MPN) contributes to immunosuppression and susceptibility to infection. Studies from a registry provided early evidence that the risk of MPN patients to die from infections may be increased when compared with normal population controls [2] and this finding was most recently underpinned by a large dataset of 8363 MPN patients [3]. MPN patients were at higher risk for severe bacterial and viral infections judged by hospital admissions and deaths from infection. The question how the type of pharmacologic treatment, the MPN subtype or the disease stage may influence the risk for infectious complications remains still unclear. Immunosuppressive effects of JAK inhibitors are determined by their specificity [4], while other cytoreductive agents such as hydroxycarbamide or interferons may also compromise the function of immune cells. Finally, molecular and clinical heterogeneity of MPN, its impact on cellular signaling, immune function and the inflammatory phenotype may vary depending on the type of driver mutation and disease burden [5, 6].

This international, patient-reported, multicenter pilot study aimed to assess for the overall incidence of infections as well as prophylactic and therapeutic measures in MPN patients in an unbiased manner. The trial included seven academic centers and two private hematology practices in two countries (Germany and Italy) and recruited between October 2018 and March 2020. The study was terminated at the onset of the CoVID-19 pandemic in Europe to avoid potential bias. Patients diagnosed with any subtype of MPN and with or without cytoreductive or symptomatic therapy were evaluated. Due to the challenge of under-reporting observed in registries or chart reviews conducted by MPN specialists [7], this pilot study was conducted as a paper–pencil based questionnaire that was completed by the patient and finally approved by the responsible physician. While this approach does not account for infections in age-matched healthy controls, duration of disease, or treatment prior to the observation period, it avoids bias for severe infections or hospitalization and includes infectious complications treated in an outpatient setting. Descriptive analysis was used to assess differences regarding the subtype of MPN, driver mutations, therapy, type, frequency and severity of infections, prophylaxis and treatment as well as assessment of chronic infections and vaccination status. For statistical comparisons, bivariate analysis by Spearman Rank Test was used when normal distribution was not given. All patients reported on the time span of 12 months prior to answering the questionnaire and independent of duration or subtype of disease.

In total, questionnaires from 948 patients with a median age of 67.0 years (range 18–99) and balanced gender distribution (425 males and 431 females) were collected. MPN subtypes included polycythemia vera (PV; 30.3%), essential thrombocythemia (ET; 40.9%), myelofibrosis (MF; 23.5%), unclassifiable MPN (MPN-U; 1.4%), and chronic myelogenous leukemia (CML; 2.7%) (Table 1). Analysis of driver mutations showed presence of *JAK2* (71.9%), *MPL* (2.7%), *CALR* (13.8%), *BCR-ABL1* (2.7%) mutations, and triple negative cases and triple-negative cases (3.4% reported as triple negative), while data on driver mutations were not reported for 51 patients (5.4%). While the majority of patients received hydroxyurea (HU) for cytoreductive or symptomatic therapy (42.9%), other treatment options included the use of the JAK1/2 inhibitor RUX (17.9%), combinations including RUX (5.6%), interferon alpha (6.0%), anagrelide (2.8%), BCR-ABL-tyrosine kinase inhibitors (TKI; 2.7%), and other agents including chemotherapy (2.6%). The majority of patients in this cohort (50.5%) reported one or more episodes of infections within the previous 12 months (Table 1). Of note, the fraction of patients reporting at least one infectious episode during the past 12 months was significantly elevated in those receiving interferon alpha, the JAK1/2 inhibitor RUX or combinations. Patients receiving no medication (55.6% ≥1 infection), HU (36.9%), or other medications (e.g. TKI; 51.1%) showed a lower frequency. Interestingly, this trend was consistent for upper respiratory tract infections and gastro-intestinal infections. As previously reported [8], herpes virus infections were most frequent in patients receiving RUX or RUX-containing combinations compared with patients receiving no medication, HU, or interferon alpha. The vast majority of patients reporting on infections did not require hospitalization (*n* = 421; 87.9%). Hospitalization occurred more frequently in patients receiving RUX or combinations (*p* = 0.05). At least one ambulatory/outpatient appointment was required in 73.8% of MPN patients with infections, with lower frequency in patients without medication (64.2%) and higher rates in patients receiving RUX or combination therapies (83.7%; *p* = 0.01).

**Table 1:** Descriptive statistics on 948 MPN patients.

	Total	Medication					
Characteristics	*n* = 948	None, *n* = 171	HU, *n* = 407	RUX, *n* = 170	Combinations, *n* = 53	Interferon, *n* = 57	Other^a^, *n* = 90
Age, years—median (range)	67.0 (18–99)	58.0 (18–86)	73.0 (18–99)	64.5 (24–88)	63.5 (39–85)	51.0 (25–80)	67.0 (20–90)
Sex							
Female—no. (%)	431 (50.4)	72 (16.7)	211 (49.0)	64 (14.8)	26 (6.0)	27 (6.3)	31 (7.2)
Male—no. (%)	425 (49.6)	79 (18.6)	82 (42.8)	62 (14.6)	20 (4.7)	25 (5.9)	57 (13.4)
MPN Subtype							
PV—no. (%)	287 (30.3)	47 (16.4)	140 (48.8)	62 (21.6)	15 (5.2)	13 (4.5)	10 (3.5)
ET—no. (%)	388 (40.9)	62 (16.0)	211 (54.4)	23 (5.9)	22 (5.7)	34 (8.7)	36 (9.3)
MF—no. (%)	223 (23.5)	54 (24.2)	48 (21.5)	82 (36.8)	14 (6.3)	10 (4.5)	15 (6.7)
MPN-U—no. (%)	24 (2.5)	7 (29.2)	8 (33.3)	3 (12.5)	2 (8.3)	0	4 (16.7)
CML—no. (%)	26 (2.8)	1 (3.8)					25 (96.2)
Driver mutation							
JAK2—no. (%)	682 (78.3)	116 (17.0)	338 (49.6)	131 (19.2)	32 (4.7)	33 (4.8)	32 (4.7)
MPL—no. (%)	26 (3.0)	9 (34.6)	9 (34.6)	2 (7.7)	1 (3.9)	2 (7.7)	3 (11.5)
CALR—no. (%)	131 (15.0)	33 (25.2)	30 (22.9)	19 (14.5)	14 (10.7)	17 (13.0)	18 (13.7)
Triple negative—no. (%)	32 (3.7)	5 (15.6)	14 (43.8)	7 (21.9)	1 (3.1)	2 (6.2)	3 (9.4)
Infections							
≥1 infection last 12 mo—no. (%)	479 (50.5)	95 (55.6)	150 (36.9)	116 (68.2)	37 (69.8)	35 (61.4)	46 (51.1)
Upper respiratory—no. (%)	338 (35.7)	72 (42.1)	106 (26.0)	83 (48.4)	20 (37.7)	23 (40.4)	34 (37.8)
Pneumonia—no. (%)	37 (3.9)	8 (4.7)	10 (2.5)	12 (7.1)	3 (5.7)	0	3 (3.8)
GI—no. (%)	135 (14.2)	33 (19.3)	18 (4.4)	40 (23.5)	15 (28.3)	15 (26.3)	12 (15.4)
Herpes virus—no. (%)	144 (15.2)	34 (19.9)	29 (7.1)	43 (25.3)	14 (26.4)	13 (22.8)	11 (12.2)
Skin—no. (%)	70 (7.4)	10 (5.8)	18 (4.4)	21 (12.4)	5 (9.4)	4 (7.0)	8 (10.3)
UTI—no. (%)	43 (4.5)	7 (4.1)	14 (3.4)	16 (9.4)	3 (5.7)	2 (3.5)	1 (1.3)
Other—no. (%)	52 (5.5)	6 (3.5)	22 (5.4)	12 (7.1)	3 (5.7)	4 (7.0)	4 (5.1)
Treatment, frequency							
Outpatient/ambulatory							
0—no. (%)	123 (25.8)	33 (35.1)	37 (24.8)	28 (24.3)	6 (16.2)	10 (28.6)	9 (19.6)
1—no. (%)	191 (40.1)	31 (32.9)	78 (52.4)	36 (31.3)	11 (29.7)	13 (37.1)	22 (47.8)
>1—no. (%)	162 (34.1)	30 (32.0)	34 (22.8)	51 (44.4)	20 (54.1)	12 (34.3)	15 (32.6)
inpatient/hospitalized							
0—no. (%)	421 (88.6)	85 (90.4)	131 (87.9)	102 (87.9)	31 (83.8)	35 (100)	0
1—no. (%)	41 (8.6)	6 (6.4)	14 (9.4)	10 (8.6)	6 (16.2)	0	0
>1—no. (%)	13 (2.8)	3 (3.2)	4 (2.7)	4 (3.5)	0	0	0
Prophylaxis							
Antibiotic—no. (%)	13 (1.4)	4 (2.3)	4 (1.0)	3 (1.8)	0	0	2 (2.2)
Antiviral—no. (%)	5 (0.5)	0	0	4 (2.4)	0	0	1 (1.1)
Antifungal—no. (%)	8 (0.8)	1 (0.6)	0	3 (1.8)	1 (1.9)	2 (3.5)	1 (1.1)
Vaccinations							
Influenza—no. (%)	364 (38.4)	41 (24.0)	175 (43.0)	70 (41.2)	26 (49.0)	18 (31.6)	34 (37.8)
Pneumococci—no. (%)	111 (11.7)	5 (2.9)	69 (17.0)	19 (11.2)	4 (7.5)	6 (10.5)	8 (8.9)
Meningococci—no. (%)	24 (2.5)	3 (1.8)	6 (1.5)	6 (3.5)	1(1.9)	5 (8.8)	3 (3.3)
Other—no. (%)	122 (12.9)	23 (13.5)	19 (4.7)	50 (29.4)	4 (7.5)	18 (31.6)	8 (8.9)
Diagnostic testing							
Hepatitis—no. (%)	147 (15.5)	25 (14.6)	27 (6.6)	53 (31.2)	15 (28.3)	17 (29.8)	10 (11.1)
Tuberculosis—no. (%)	81 (8.5)	17 (9.9)	13 (3.2)	30 (17.6)	8 (15.0)	9 (15.8)	4 (4.4)
Toxoplasmosis—no. (%)	69 (7.3)	13 (7.6)	10 (2.5)	26 (15.3)	6 (11.3)	10 (17.5)	4 (4.4)

*mo* months, *no*. number, *PV* polycythemia vera, *ET* essential thrombocythemia, *MF* myelofibrosis, *MPN-U* MPN unclassifiable, *CML* chronic myelogenous leukemia, *GI* gastro-intestinal, *UTI* urinary tract infection, *HU* hydroxyurea, *RUX* ruxolitinib.

^a^Other: TKI; chemotherapeutic agents; anagrelide. Combination therapies included RUX plus either agent: hydroxyurea, pomalidomide, MDM2-inhibitors, BET-inhibitors, or interferon alpha.

Recommendations of the German Standing Committee on Vaccinations (STIKO) include vaccinations against influenza, herpes zoster, and pneumococci for individuals beyond the age of 60 and meningococci for those with a preexisting comorbidity of the immune system [9]. Current MPN treatment guidelines do not generally recommend prophylactic use of antibiotics, antiviral, or antifungal medications [10]. However, recommendations of the European Medicines agency exclude patients with chronic virus infections (such as hepatitis B) from RUX treatment [11]. Consistently, expert opinions published on the immunosuppressive effects of RUX recommended diagnostic testing for chronic infections such as hepatitis and tuberculosis, as prophylactic measures are available and safe [12]. In this study, assessment for prophylactic medications or vaccinations showed that only a minority of patients reported on the use of antibiotic (1.4%), antiviral (0.5%), or antifungal (0.8%) prophylaxis (Table 1). Notably, most patients had not been tested for tuberculosis (91.5%), hepatitis (84.5%), or toxoplasmosis (92.7%) prior to MPN therapy. This finding is of utmost interest taking into account the potential complications in case of disease reactivation and the rate of patients diagnosed with tuberculosis (1.4%), hepatitis (3.3%), or toxoplasmosis (0.8%), if tested before treatment initiation (Table 1). The number of patients that were tested for preexisting chronic infections appears rather low, especially when compared with 24.5% of patients treated with JAK inhibitors or combinations, who can experience relevant T-cell suppression [13]. Also, the minority of patients had received vaccinations for influenza (38.4%), pneumococci (11.7%), meningococci (2.5%), or other vaccines (12.9%) including those against herpes zoster, in an elderly population at risk with a median age of 67 years.

Consistent with previous reports, diagnosis of MF (57.4% ≥1 infection; *p* = 0.022) as well as JAK inhibitor (RUX) treatment (68.2% ≥1 infection; *p* = 0.01) resulted in a significantly increased risk of infections (Table 2). Even more pronounced patients receiving combinations including RUX were at higher risk in bivariate (69.8% ≥1 infection; *p* = 0.04; Table 2) and multivariate analysis (odds ratio 1.307; 95% Wald CI: 0.860–4.327; *p* = 0.042). Although the number of interferon alpha treated patients is relatively low, the rate of viral infections appears relevant. Unexpectedly, patients on HU had a lower risk for infectious complications than patients without pharmacologic treatment or interferon therapy. This may be explained by its predominant use in early PV and ET and the fact that 54% of patients without any treatment are diagnosed with more severe MPN subtypes such as MF and therefore more prone to viral infection. Driver mutations showed no influence on the risk of infection (Table 2). Interestingly, older patients above the age of 65 (that were otherwise represented in all disease subtypes and treatment modalities) showed lower risk of infections in bivariate (*n* = 483; *p* = 0.01; Table 2) and multivariate analysis (odds ratio 1.627; 95% Wald CI: 1.192–2.221; *p* = 0.002).

**Table 2:** Clinical characteristics of MPN according to the occurrence of infectious events.

Characteristics	Total	No Infection	Infection	*p*
Age				
>65 years—no. (%)	483 (51.0)	287 (59.4)	196 (40.6)	0.01
MPN subtype				
PV—no. (%)	287 (30.3)	144 (50.2)	143 (49.8)	0.75
ET—no. (%)	388 (40.9)	204 (52.6)	184 (47.4)	0.11
MF—no. (%)	223 (23.5)	95 (42.6)	128 (57.4)	0.022
MPN-U—no. (%)	24 (2.5)	12 (50.0)	12 (50.0)	0.93
CML—no. (%)	26 (2.8)	13 (50.0)	13 (50.0)	0.92
Driver mutation				
JAK2—no. (%)	682 (78.3)	342 (50.1)	340 (49.9)	0.45
MPL—no. (%)	26 (3.0)	13 (50.0)	13 (50.0)	0.93
CALR—no. (%)	131 (15.0)	62 (47.3)	67 (52.7)	0.64
Triple negative—no. (%)	32 (3.7)	13 (40.6)	19 (59.4)	0.32
Therapy				
No medication—no. (%)	171 (18.0)	76 (44.4)	95 (55.6)	0.12
HU—no. (%)	407 (42.9)	257 (63.1)	150 (36.9)	0.01
Ruxolitinib—no. (%)	170 (17.9)	54 (31.8)	116 (68.2)	0.01
Combination—no. (%)	53 (5.6)	16 (30.2)	37 (69.8)	0.04
Interferon—no. (%)	57 (6.0)	22 (38.6)	35 (61.4)	0.09
Other—no. (%)	90 (9.5)	44 (48.9)	46 (51.1)	0.94

Combination therapies included RUX plus either agent: hydroxyurea, pomalidomide, MDM2-inhibitors, BET-inhibitors, or interferon alpha.

*no*. number, *PV* polycythemia vera, *ET* essential thrombocythemia, *MF* myelofibrosis, *MPN-U* MPN unclassifiable, *HU* hydroxyurea.

To the best of our knowledge this is the largest patient-reported assessment of infectious complications and prophylactic measures in MPN patients. While this exploratory pilot trial is limited in its assessment of comorbidities and global immune function of the individual patient, it provides first evidence for the overall incidence of infections irrespective of pharmacologic therapy, MPN subtype, severity of disease (hospitalization or ambulatory) and nature of medical care (practice based hematology or academic center). The results emphasize the need to test for preexisting chronic infections and to discuss preventative measures such as consequent use of vaccines independent of age. Therefore, this study may be clinically meaningful in the light of approval and clinical use of established and novel JAK inhibitors that confer immunosuppression and increase the risk for virus infection and reactivation. Along these lines it is tempting to speculate on improved risk control when strictly testing for preexisting infections, vaccinating those at risk according to existing recommendations and using pharmacologic prophylaxis where appropriate.
